# From benign to pathogenic variants and vice versa: pyrimidine transitions at position -3 of TAG and CAG 3*'* splice sites

**DOI:** 10.1038/s10038-024-01308-8

**Published:** 2024-12-05

**Authors:** Igor Vořechovský

**Affiliations:** Faculty of Medicine, HDH, University of Southampton, Southampton, United Kingdom

**Keywords:** Gene expression, Genetics research

## Abstract

In the human genome, CAG 3*'* splice sites (3*'*ss) are more than twice as frequent as TAG 3*'*ss. The greater abundance of the former has been attributed to a higher probability of exon skipping upon cytosine-to-thymine transitions at intron position -3 (-3C > T) than thymine-to-cytosine variants (-3T > C). However, molecular mechanisms underlying this bias and its clinical impact are poorly understood. In this study, base-pairing probabilities (BPPs) and RNA secondary structures were compared between CAG 3*'*ss that produced more skipping of downstream exons than their mutated UAG versions (termed “laggard” CAG 3*'*ss) and UAG 3*'*ss that resulted in more skipping than their mutated CAG counterparts (canonical 3*'*ss). The laggard CAG 3’ss showed significantly higher BPPs across intron-exon boundaries than canonical 3*'*ss. The difference was centered on positions -5 to -1 relative to the intron-exon junction, the region previously shown to exhibit the strongest high-resolution ultraviolet crosslinking to the small subunit of auxiliary factor of U2 snRNP (U2AF1). RNA secondary structure predictions suggested that laggard CAG 3*'*ss were more often sequestered in paired conformations and in longer stem structures while canonical 3*'*ss were more frequently unpaired. Taken together, the excess of base-pairing at 3*'*ss has a potential to alter the hierarchy in intrinsic splicing efficiency of human YAG 3*'*ss from canonical CAG > UAG to non-canonical UAG > CAG, to modify the clinical impact of transitions at this position and to change their classification from pathogenic to benign or vice versa.

## Introduction

Prediction of phenotypic consequences of somatic and germline mutations or natural DNA polymorphisms is a major challenge in biology and medicine. Despite considerable resources dedicated to this field, only a small fraction of sequence variants described to date has been associated with altered interactions of variant alleles with cellular components. The number of ‘likely benign’, ‘likely pathogenic’ or ‘variants of uncertain significance’ deposited in multiple databases has risen dramatically in the last decades [1]. As a result, the translational potential of genomics has remained limited by our inability to reliably predict which variants lead to actionable phenotypes, often prohibiting accurate diagnosis and counseling. This challenge is magnified by realization that even identical mutations at the same position of traditional splice-site consensus sequences, such as those discussed in this work, may have unexpected or even opposite phenotypic effects, depending on their genomic context.

Splicing outcomes can be affected by formation of RNA structures by nascent transcripts and can be modified even by a single-nucleotide change [2–11]. RNA secondary structures may inhibit or promote RNA processing or even replace a need for protein splicing factors (reviewed by [11]). Their formation involves both the traditional splicing signals (splice sites, branch points and polypyrimidine tracts), for example by bringing adjacent splice sites to proximity [5], and the auxiliary splicing motifs in exons [6, 12]. However, the importance of intramolecular RNA base-pairing at individual splice-site positions is poorly understood.

A striking example of the functional dichotomy of splice-site mutations are pyrimidine transitions at position -3 relative to intron-exon junctions. This position forms a part of the 3*'* splice site (3*'*ss) consensus sequence YAG/G (where Y is cytosine or uridine and / is the intron-exon boundary). During evolution, nucleotide preferences at position -3 coevolved with increasingly more complex spliceosomes: whereas many yeast species prefer uridines ([13] and refs. therein), mammalian 3*'*ss generally favor cytosines while other lineages such as some nematodes have almost exclusively cytosines ([14–17] and refs. therein). As an example, *Caenorhabditis elegans* 3*'*ss are defined by a highly conserved octamer UUUUCAG/R where -3C must remain adjacent to the AG/R motif for efficient splicing [16]. In the human genome, the number of CAG 3’ss exceeds TAG 3’ss by a factor of ~2.2 [14, 18]. The CAG and TAG 3*'*ss were initially regarded as functionally equivalent and transitions -3T > C or -3C > T as splicing-neutral [19–21]. However, increasing numbers of reports have now convincingly shown that mutations -3C > T do not only induce exon skipping [18, 22–25], but also promote exon inclusion [18, 26, 27] and/or activate silent or cryptic 3’ss [18, 28]. Testing a large number of human exogenous transcript pairs clearly showed that when CAG or UAG 3*'*ss of minigene mid-exons were cloned between identical exons and intronic segments, exon skipping was a preferred outcome for mutations -3C > T while mutations -3T > C usually improved exon inclusion [18]. A small number of inefficient CAG 3*'*ss (termed here ‘laggard’ 3*'*ss) do not obey the accepted trend that -3T > C transitions are typically less detrimental than -3C > T transitions [18, 28]. It remains unclear, however, if this bias can explain the higher abundance of CAG 3*'*ss in mammalian genomes. In addition, no ab initio tools exist to identify anomalous YAG 3*'*ss that increase exon skipping when mutated from UAG to CAG. Finally, it has been unclear why the non-canonical -3T alleles can, in some cases, promote exon inclusion as compared to the -3C alleles and thus become superior to canonical -3C alleles.

To address the last question, this study has compared base-pairing probabilities (BPPs) of transcript pairs with laggard CAG 3*'*ss and canonical 3*'*ss. Even the small number of informative transcript pairs (*n* = 22) has revealed higher average BPPs across intron-exon junctions of laggard CAG 3*'*ss (ie. 3*'*ss with the hierarchy in splicing efficiency of UAG > CAG) as compared to 3*'*ss with the canonical order CAG > UAG. The maximum discrimination was observed for positions -5 to -1 relative to 3*'*ss, consistent with the involvement of U2AF1. These results suggest that the accessibility of pyrimidine bases at position -3 can control not only splicing efficiency but also clinical outcome of these mutations on a scale benign to pathogenic or vice versa.

## Materials and methods

### Nucleotide sequences

Sequences of primary transcripts with CAG 3*'*ss that preferred exon skipping (laggard CAG 3*'*ss) as compared to UAG 3’ss counterparts are shown at the top of Table 1. Sequences of primary transcripts with UAG 3*'*ss that preferred exon skipping as compared to CAG 3*'*ss counterparts are shown at the bottom of Table 1. The transcripts were identified previously in our study of >80 minigene constructs [18], which were mostly derived from established human disease genes that sustained transitions –3 C > T or –3 T > C implicated in the phenotype. Disease genes, clinical phenotypes, splicing patterns and nucleotide sequences were compiled in Supplementary Table S5 of ref. [18]. The splicing patterns of minigene constructs were shown in Fig. 4 of the same reference. Each mutated 3*'*ss sequence was confirmed by Sanger sequencing of plasmid constructs [18] and was identical to reference sequences of human genes [29].

**Table 1:** Nucleotide sequences of 3*'* splice sites in 22 informative transcript pairs and exon skipping preferences of their -3C/T alleles

Gene	Mutation	3*'*ss that prefers ES^a^	RNA sequence across 3*'*ss^b^	Conformation at -3C/-3U^c^	ES (%) of -3C:-3U^d^
*EPS15*	c.652-3 T/C	CAG	UACUGUUUUUUUUCCUCCCUGCAG/UGGGUUGUAUC	P/Pw	7:0
*ABCA4*	c.5899-3 T > C	CAG	GGCUAGCUCUGUGUUUUCUCCCAG/UGCUUUGGCCU	P/Pw	46:0
*CAPS*	c.84-3 T/C	CAG	UCCAACCGUGUCCCCUGCCUCCAG/GUUUUUCCGCC	P/P	40:28
*UHRF1*	c.887-3 T/C	CAG	CUGACCCUGCCGCCCCGUGCCCAG/GGAAGAGCGGG	P/B	80:21
*CXXC1*	c.460-3 C/T	UAG	UCUGUUCUGGGCCCCCUCCUGUAG/CAUCACCAGCA	P/Pw	1:5
*SRI*	c.249-3 C/T	UAG	CUCUAAUCCUUGAUUACAGUUUAG/AGAGAUAUGUC	P/P	79:96
*NOX5*	c.175-3 C/T	UAG	GUCUUCCACCCUUCUCGCCCAUAG/UCCUUCUUUGC	U/U	4:16
*CRACR2A*	c.229-3 C/T	UAG	CAGUACACUCUGGGUUGUUUUUAG/AGGCUGCAUAA	U/U	2:7
*HGD*	c.650-3 C/T	UAG	GACUUUUGGGUUACUGUUUUCUAG/GGGCCAAUGGC	P/U	0:5
*F8*	c.5999-3 C/T	UAG	UUCUUCACUGUCCCUUUAAAAUAG/AUUUGGCCAGG	P/P	4:22
*UBE2F*	c.215-3 T/C	UAG	GUUUUGUUUUGUGUUUUUUGAUAG/AUGAGGGUUAC	P/U	21:70^f^
*SMN1*	c.835-3 T/C	UAG	UAACUUCCUUUAUUUUCCUUAUAG/GGUUUCAGACA	U/U	0:27
*OTC*	c.867-3 T > C	UAG	GUGGUCUUAUCCCCAUCUCUUUAG/ACUGCUAAAGU	U/P	12:93
*PKHD1*	c.2141-3 T > C	UAG	AGUAAUUGGAUCACUGGUCUCUAG/UUUCUCAAGCU	P/U	0:9
*SGCE*	c.391-3 T > C	UAG	AAUAUGGUUUUCCUUUUAUAAUAG/AUAACUGCCUA	U/U	0:4
*CFTR*^*e*^	c.165-3 C > T	UAG	GUCCCACUUUUUAUUCUUUUGUAG/AGAAUGGGAUA	U/P	0:24
*CRB2*	c.941-3 C > T	UAG	GACCCACAGCUGGGCCUCUUAUAG/GAGCCGACUGC	U/U	17:83
*FRMD7*	c.498-3 C > T	UAG	CUCAUAAAUUCUUUCCCCUUUUAG/UGGCAGGAGCC	P/Pw	0:3
*KIF5A*	c.2993-3 C > T	UAG	UGUUCUCAAUGAUGAUCUCUUUAG/GAAAUGCCACA	U/U	21:100
*NIPBL*	c.3856-3 C > T	UAG	UUCAUUAACAAUACUGUUUUAUAG/AAUAACGAUAC	P/P	4:100
*PARN*	c.178-3 C > T	UAG	AGAUGUUUUAUUUCCCUUUUCUAG/CAUUCCAUGGA	U/U	12:34
*AMELX*	c.103-3 T > C	UAG	UUUACCUUCUUCUUUCUUUUGUAG/AACUCACAUUC	U/U	13:98

^a^CAG or UAG 3*'*ss that showed higher exon skipping (ES) than their mutated versions in 22 tested minigene pairs (Fig. 1a). The sequence of minigene pairs differed only by pyrimidine at position -3 relative to 3*'*ss of the middle exon. Only the less efficient allele is shown per transcript pair. CAG 3*'*ss that showed higher ES than their UAG 3*'*ss counterparts (laggard CAG 3*'*ss) are at the top (n = 4); UAG 3*'*ss that showed higher ES than CAG 3*'*ss counterparts are at the bottom (n = 18)

^b^Intron-exon junctions are denoted by a slash; position -3 is underlined. Compilation of all disease-associated pyrimidine-to-pyrimidine mutations at position -3 as and their wild-type sequences are in Tables S5 and S6 of ref. [18]

^c^Paired (P), wobble (Pw), bulged (B), and unpaired (U) conformation of -3C and -3U bases in the most stable minimum free energy models

^d^Exon skipping (%) of the two alleles (CAG:UAG) was measured previously [18]

^e^CFTR refers to CFTR exon 3 in ref. [18]

^f^Exon skipping was induced only by weakening both 3’ss with a mutated PUF60 [18]

### Computing base-pairing probabilities

BPPs for individual RNA sequences were defined previously [30]. Briefly, a BPP *p*_*ij*_ (*i* < *j*) is equal to the probability that the *i*th and *j*th nucleotides of a sequence form a base pair and can be interpreted as a confidence measure of predicted base pairs. BPPs were computed using CentroidFold [31], employing both CONTRAfold [32] and McCaskill [33] algorithms.

### RNA secondary structure predictions

Minimum free energy and centroid predictions were carried out using RNAfold and Centroid [31, 34]. Unlike the minimum free energy, which is regarded as a maximum likelihood estimator, the Centroid estimator considers the entire distribution over the solutions instead of only the solution with the highest probability [35]. Paired, wobble and unpaired configuration at position -3 was recorded for each transcript in both groups of 3’ss using most stable minimum free energy and Centroid predictions (Table 1, Supplementary Fig. S1).

### PU values

PU (probability of unpaired) values were computed for 100 nucleotides covering 3*'*ss and 30 nucleotides of flanking sequences in each direction, as described [6, 36]. Briefly, the PU value for the region *a* to *b* in an RNA sequence is defined as *e*^([*E*_all _− *E*_unpaired_]/RT), where *E*_all_ is the free energy of the ensemble of all structures, *E*_unpaired_ is the free energy of the ensemble of all structures that have the complete region *a* to *b* unpaired, *R* is the universal gas constant, and *T* is the temperature [6]. *E*_all_ and *E*_unpaired_ values were computed using RNAfold [34]. PU values were also implemented in the NIPU server at http://rna.informatik.uni-freiburg.de/NIPU/Input.jsp [6]. PU values range between 0 (completely base-paired) and 1 (completely unpaired).

### Statistical analysis

BPP and PU values were averaged and means and standard deviations of the two groups of 3*'*ss were compared using an unpaired t-test. Nucleotide distribution across 3*'*ss and distribution of paired and unpaired nucleotides in most stable structures was compared using *χ*^2^ tests.

## Results

**Fig. 1:**
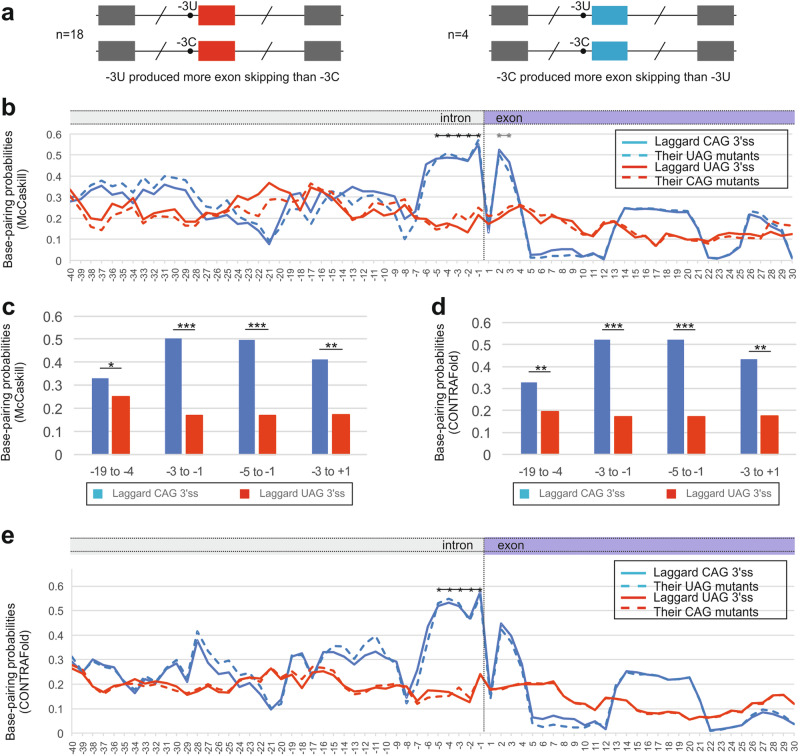
**CAG 3*'*** **splice sites that produce more exon skipping than their UAG counterparts are associated with higher base-pairing probabilities across introns-exon boundary. a** Schematics of plasmid construct pairs (*n* = 22). Each minigene had identical first and third exons. The tested exon in the middle had either CAG or UAG 3*'*ss, but no other sequence changes [18]. **b** Average BPPs across laggard and canonical 3*'*ss (*n* = 4 and 18, respectively) and their allelic counterparts. Native 3*'*ss are shown as solid lines, dashed lines represent BPP values for alternate pyrimidines. Asterisks represent the region with significant differences between the two groups of 3*'*ss. **c, d** Mean BPP values for the indicated regions and associated *P*-values for McCaskill (**c**) and CONTRAFold (**d**) algorithms. *P value < 0.05, ***P < 0.0001 (unpaired t-tests). **e** Mean BPPs across laggard and canonical 3*'*ss and across their allelic counterparts, as computed using CONTRAFold

Figure 1a summarizes tested minigene transcript pairs. Each pair had two transcript versions that differed only by a pyrimidine at position -3 of 3*'*ss of middle exons. By screening over 40 minigene pairs containing transitions -3C > T or -3T > C, 22 pairs were informative (Table 1 and ref. [18]). They consisted of 18 pairs where UAG 3*'*ss generated more exon skipping than their CAG 3*'*ss counterparts and 4 pairs where CAG 3’ss produced more exon skipping than UAG 3*'*ss counterparts [18]. The screening indicated that human -3T > C transitions are usually, but not always, less detrimental than -3C > T transitions and identified a small group of non-canonical, ‘laggard’ CAG 3*'*ss that produced lower exon inclusion in mature transcripts than their mutated UAG versions (Table 1).

Figure 1b shows BPPs for the two groups of 3’ss and their allelic variants with alternate pyrimidines at position -3. Using the McCaskill algorithm [33], the laggard CAG 3’ss had higher BPPs at positions -5 to -1 than 3*'*ss that promoted exon skipping upon transitions -3C > T (Fig. 1b, c). This tendency was also observed for exon positions +2 and +3, but not for the first exon nucleotide. CONTRAfold [32] identified a similar increase for laggard CAG 3*'*ss although the exonic peak was lower (Fig. 1d, e). BPPs of the same transcripts mutated at position -3 to the other pyrimidine maintained higher values at positions -5 to -1 (Fig. 1b–e).

**Fig. 2:**
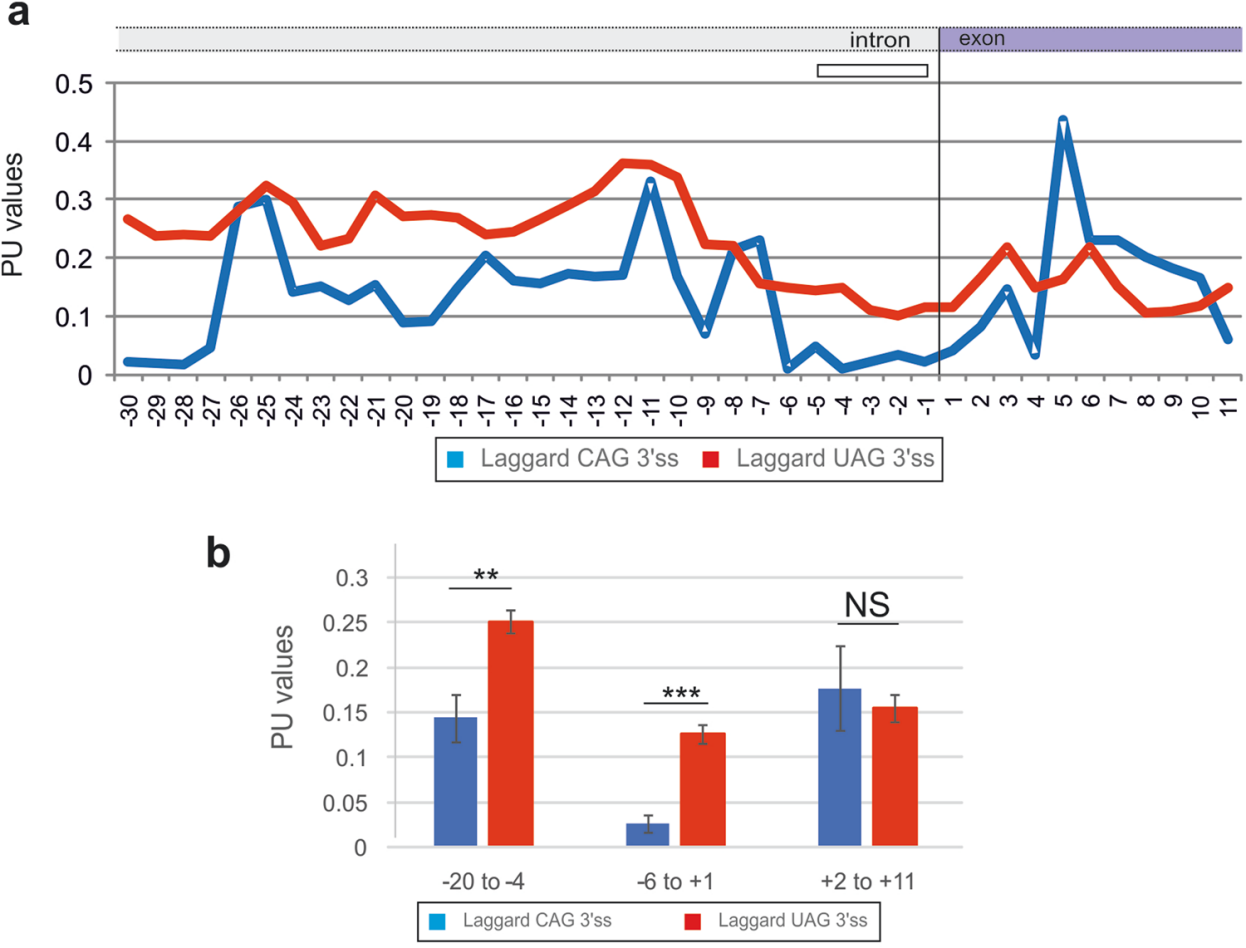
**PU values across laggard and canonical 3*'*** **splice sites**. **a** Mean PU values across 3*'*ss sequences of the two groups of 3*'*ss. **b** Comparison of average PU values for the indicated positions relative to the intron-exon junction (vertical line). **P value < 0.001, ***P < 0.0001 (unpaired t-tests). NS not statistically significant

The distinct BPP profiles between laggard CAG 3*'*ss and canonical CAG 3*'*ss were also found with a measure of RNA single-strandedness computed as the probability that all bases in the sequence are unpaired (termed PU values) [6]. The average PU values are elevated for auxiliary splicing motifs in exons that promote exon inclusion in mature transcripts [6]. PU profiles across laggard CAG 3*'*ss showed low values between position -5 and +2, consistent with their higher CONTRAFold- and McCaskill-derived BPPs (Fig. 2a). A decrease of PU values could be seen also further upstream (Fig. 2a, b).

**Fig. 3:**
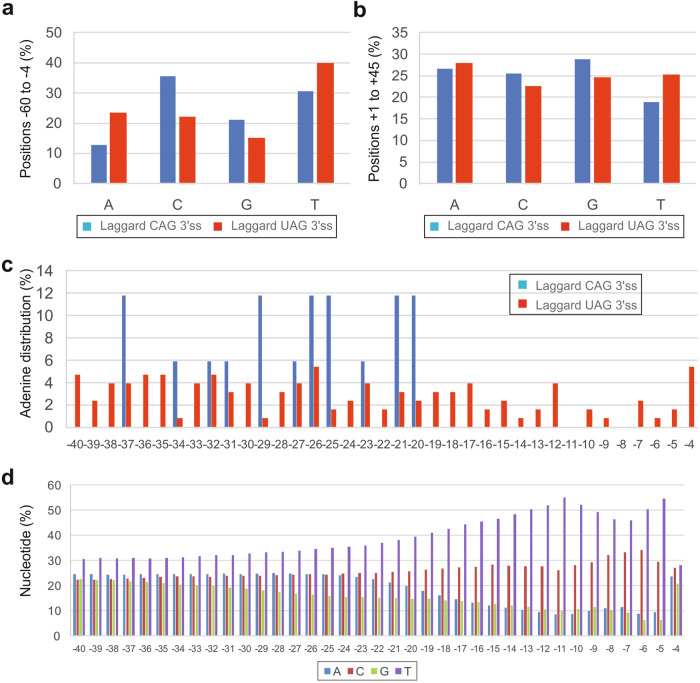
**A lack of adenines and uridines between positions -3 and -20 of laggard CAG 3*'*** **splice sites**. **a**, **b** A lack of adenines and, to a lesser degree, uridines upstream (**a**) but not downstream (**b**) of the intron-exon boundary. *χ*^2^ values for 2 × 4 contingency tables were 31.7 (*P* < 0.0001) (**a**) and 4.2 (*P* = 0.2) (**b**). **c** Adenines were absent just upstream of laggard CAG 3*'*ss. **d** Nucleotide distribution upstream of 195,404 human 3’ss

Nucleotide distribution of the two groups of 3*'*ss showed that laggard CAG 3*'*ss lacked adenines and, to a lesser extent, uridine upstream (Fig. 3a) but not downstream (Fig. 3b) of 3*'*ss. At positions -19 to -3 relative to the intron-exon junction, adenine was completely absent (Fig. 3c), although this could result from a chance since adenine is depleted in this region of human introns (Fig. 3d). Nevertheless, the altered nucleotide composition of laggard 3*'*ss is likely to influence secondary pre-mRNA structures formed during or after transcription by reducing weaker A:T base-pairing and increasing stronger C:G base-pairing, consistent with their higher BPPs (*cf*. Figs. 1b and 2a).

**Fig. 4:**
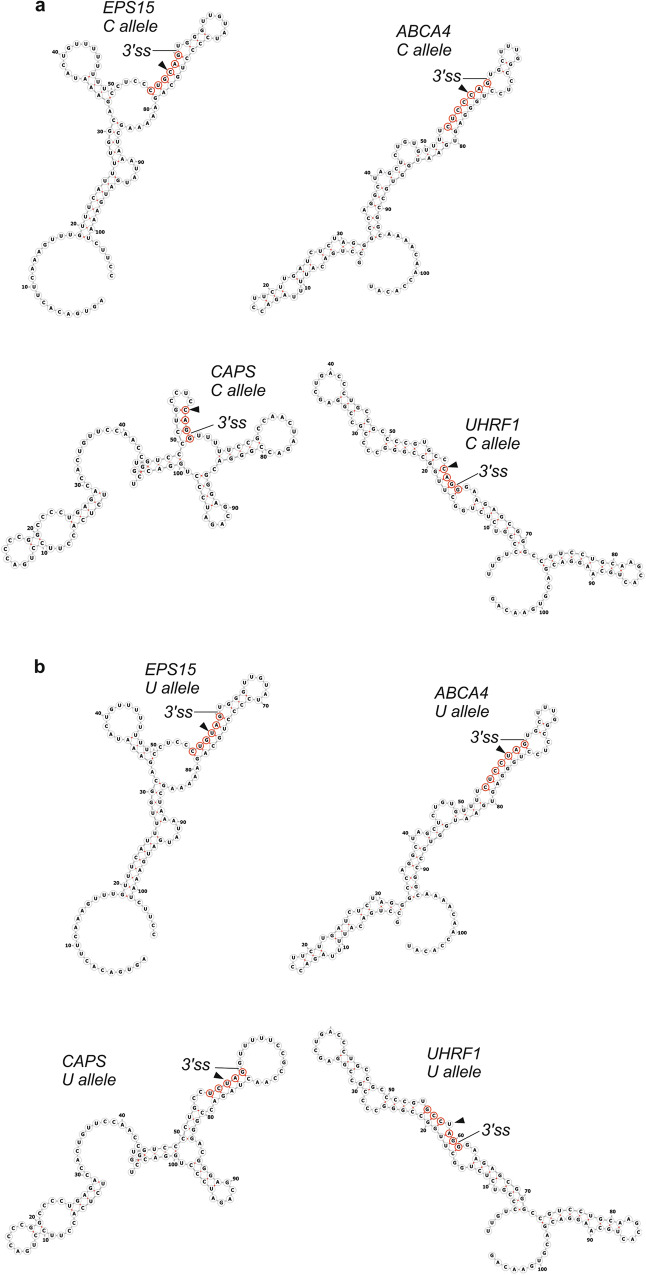
**Laggard CAG 3*'*** **splice sites may require longer stem structures across intron-exon junctions**. **a** Transcripts with cytosine alleles. **b** Transcripts with uridine alleles. Secondary structure predictions were carried out by RNAfold [34, 50]. Predicted structures of canonical (laggard UAG) 3’ss are shown in Supplementary Fig. S1

Figure 4a shows examples of most stable local structures for laggard CAG 3*'*ss. In *EPS15*, the transcript with the highest average BPP at positions -5 to -1 (0.98), a six base-pair stem sequestering 3*'*ss was further extended by three consecutive C:G pairs at exon positions +2 through +4. In *ABCA4*, the transcript with the second highest BPPs in this region (0.85), the 3*'*ss is sequestered in a continuous seven base-pair stem. In both transcripts, the stems involving 3*'*ss are more stable with the C allele as opposed to the U allele (Fig. 4b) and their length is close to a threshold required for rapid annealing of DNA or RNA [37]. The remaining transcript pairs where UAG 3’ss were superior to CAG 3*'*ss had shorter stems but the stems included the full YAG 3*'*ss consensus in each case. In each laggard CAG 3*'*ss, the hairpin structures had either four- or six-nucleotide loops; loops of the same size consistently inhibited splicing in *Saccharomyces cerevisiae* when introduced at 5*'*ss and branch point regions [4]. In contrast, RNA secondary structure predictions for eighteen 3*'*ss that preferred canonical CAG showed incompletely paired YAG 3*'*ss motifs at position -3 in 11 (50%) cases (*χ*^2^ = 5.5, *P* = 0.02; Table 1 and Supplementary Fig. S1). The higher frequency of paired interactions involving 3*'*ss consensus in laggard CAG 3*'*ss than canonical 3*'*ss suggests that if -3C is paired, laggard CAG 3*'*ss could occur more likely than in the unpaired context.

In conclusion, independent profiling of BPPs and PU values across the two groups of 3*'*ss identified significant increase in predicted base-pairing in the group of transcripts where UAG 3*'*ss were, atypically, more efficient than their CAG 3*'*ss versions. Therefore, RNA secondary structure has a potential to alter the hierarchy in intrinsic efficiency of human 3’ss from canonical CAG > UAG(>AAG > GAG) to non-canonical UAG > CAG(>AAG > GAG) (3*'*ss in parentheses have not been tested in this work). As a result, the same C > T or T > C mutations at position -3 of 3*'*ss can have distinct phenotypic outcomes in different sequence and structural contexts.

## Discussion

### Becoming laggard CAG 3*'* splice sites

This study shows that human CAG 3*'*ss that include downstream exons less efficiently in mature transcripts than their UAG 3*'*ss counterparts are more structured across intron-exon boundaries and suggests that prediction of phenotypic outcomes of pyrimidine transitions at position -3 could be improved by considering intramolecular base-pairing. In other words, the higher BPPs across laggard CAG 3*'*ss can reduce their splicing efficiency to the extent that they become worse performers than the UAG versions of the same transcripts. This would explain why a subset of -3T > C transitions in human disease genes can, atypically, reduce exon inclusion [18, 26, 27], which may lead to a more frequent disease occurrence, earlier onset, faster disease progression and/or greater severity. However, it remains to be tested whether the -3C/T transitions are more likely to be functional if the secondary structure of the pre-mRNA is permissive, ie. less paired around 3*'*ss (Table 1 and Supplementary Fig. S1).

The BPP differences between the two groups of 3*'*ss were centered on intron positions -5 to -1 (Fig. 1b–e). Given the small number of laggard CAG 3*'*ss identified so far, it cannot be excluded that altered BPP and/or PU values at these 3*'*ss extend to other exon or intron positions or splicing motifs, such as branch point sequences and polypyrimidine tracts (Figs. 1b, e, and 2a). The 22 transcript pairs examined in this study were identified by screening >40 minigene pairs with -3 C > T or -3T > C transitions, indicating that about a half of the pairs were not informative, ie. with zero or 100% exon inclusion for each allele or identical exon inclusion levels [18]. Such 3*'*ss may still display -3C/T allelic differences in splicing efficiency, which could be unmasked by 3*'*ss weakening [18]. These figures suggest that identification of one additional laggard CAG 3*'*ss would require examination of about 10 more transcript pairs in transfection studies. The fraction of human 3’ss with the canonical order CAG > UAG > AAG > GAG in splicing efficiency is thus unlikely to exceed 90% and is probably lower than that. Because the number of -3C > T or -3T > C transitions in patients with genetic disorders is growing, approaching a hundred of reported cases (compiled in ref. [18]), the expansion of this study to obtain a larger dataset should be feasible in the future. The sample expansion could be facilitated by focusing on -3C/T variants incontrovertibly associated with QTLs, ultimately providing more robust evidence and improving ab initio predictions of laggard CAG 3*'*ss. At present, BPP values alone are unlikely to provide sufficient discrimination power to distinguish benign and pathogenic variants. Moreover, it remains unclear to what extent a relatively small depletion of uridines upstream of laggard CAG 3*'*ss (Fig. 3a), potentially reducing U2AF interactions, rather than secondary structure constraints could switch CAG *versus* UAG 3*'*ss preferences in splicing efficiency.

Although hairpin structures can influence splice site selection from yeasts to humans [3, 4, 9, 10, 38], it has not been possible to reliably predict without testing whether a particular structure impedes or promotes exon inclusion. Four- or six-nucleotide loop hairpins sequestering 5*'*ss and branch points always inhibited RNA splicing in vivo and in vitro [4], consistent with loop sizes predicted for laggard CAG 3*'*ss (Fig. 4), but only rarely for canonical (laggard UAG) 3*'*ss (Supplementary Fig. S1). Establishing a larger group of laggard CAG 3’ss and their local folding patterns should help define molecular interactions at this position and 3*'*ss responses to dynamic secondary structure formation across intron-exon junctions.

As compared to position -3, anomalous behavior of C/T variants is less well documented in polypyrimidine tracts, which are usually located between lariat intron branch points and position -5. Here, uridines are preferred over cytosines, particularly in shorter polypyrimidine tracts [39]. Although unpublished or anecdotal findings suggest that this is not always the case, systematic studies of the splicing impact of C/T transitions at this location have not been available. Identification of anomalous mutations in these upstream locations in genetic disease is likely to be more arduous than anomalous mutations at position -3 of 3*'*ss since upstream transitions may often lead to less significant splicing alterations and only mild or low-penetrance phenotypes [40]. Their pathogenicity could be merely manifested by an overrepresentation of cytosines over uridines in disease phenotypes as compared to controls, as suggested for pheochromocytomas [41]. The milder character of C/T transitions in polypyrimidine tracts could reflect structural preferences of the large subunit of U2AF (U2AF2) for pyrimidines and for hydrogen bonds of uracil edges as opposed to sequence-specific recognition strategies of other interacting regulators, such as PTB or SXL [42].

### The role of U2AF in generating laggard CAG 3*'*ss

Static models of RNA secondary structure suggest that position -3 accessibility may expose intrinsic binding differences between CAG and UAG 3*'*ss: if -3 positions are paired, differences between -3C and -3U binding to *trans*-acting factors could be masked (Fig. 4). The hottest candidate for this interaction is the small subunit of U2AF, or U2AF1, which forms a heterodimer with U2AF2 and contacts 3*'*ss early during spliceosome assembly [43–46]. In high resolution ultraviolet crosslinking and immunoprecipitation studies with the wild-type, U2AF1 showed strongest signals for terminal five nucleotides of the introns [47]. This preference was maintained for U1AF1 with a cancer-associated substitution S34F, but S34F shifted the most frequently crosslinked nucleotide by a single position and altered pyrimidine distribution at position -3 in favor of CAG 3*'*ss [47]. -3C was the most common nucleotide preceding S34F-promoted exons in independent studies [48]. For yeast U2AF1, U*C*AG*N*U RNAs had consistently higher dissociation constants and weaker binding in isothermal titration calorimetry studies than U*U*AG*N*U oligomers (where italics denote variant positions and *N* is any nucleotide) [49], but uridine is preferred in yeasts, unlike in humans [17]. The binding preferences of yeast U2AF1 to 3*'*ss were also affected by substitution S34F [49], nevertheless comparable calorimetry data for human U2AF1 complexed with a short fragment of U2AF2 have not been available. Apart from U2AF subunits, one cannot exclude other interactions between the same pre-mRNA segment and small nuclear RNAs or proteins. The reported preference in binding of wild-type U2AF1 to UAG 3*'*ss [43–47, 49] would not explain the canonical order in the efficiency of human 3*'*ss usage (CAG > UAG > AAG > GAG) [18, 19].

Finally, the variable outcome of -3C/T transitions supports a speculation that the selection pressure at position -3 has been relatively mild during recent evolution, but could be stronger earlier, such as during evolution of single-cell eukaryotes. Distribution of pyrimidines at position -3 did not appear to play a role in compensatory responses of human traditional or auxiliary splicing motifs when comparing highly conserved exons encoding calcium- and zinc-coordinating residues in metalloproteins [18].

In conclusion, if more structured, the more abundant and generally more splice-proficient CAG 3*'*ss may turn into “laggards” and skip the downstream exon more than their intrinsically weaker UAG 3’ss counterparts. This work identifies a collection of 3*'*ss that provide a starting point for exploring structural requirements for their usage in much greater detail, which should facilitate our understanding of structural interactions that involve position -3. These results also suggest that prediction of splicing and clinical outcomes of DNA mutations and polymorphisms in mammalian genes may never be 100% accurate without considering RNA structure of primary transcripts, particularly across traditional and auxiliary splicing motifs.

## Supplementary information

Supplemental Fig. S1

## Data Availability

The data generated or analyzed during this study can be found within this article and its supplementary file.
