# Supplementary material for: From benign to pathogenic variants and vice versa: pyrimidine transitions at position -3 of TAG and CAG 3' splice sites
Source: J Hum Genet. 2024 Dec 5;70(3):125–33. doi: 10.1038/s10038-024-01308-8 (PMC11802449; doi:10.1038/s10038-024-01308-8)
Supplement: Supplementary file 1 — Supplemental Fig. S1 [file 10038_2024_1308_MOESM1_ESM.pdf]

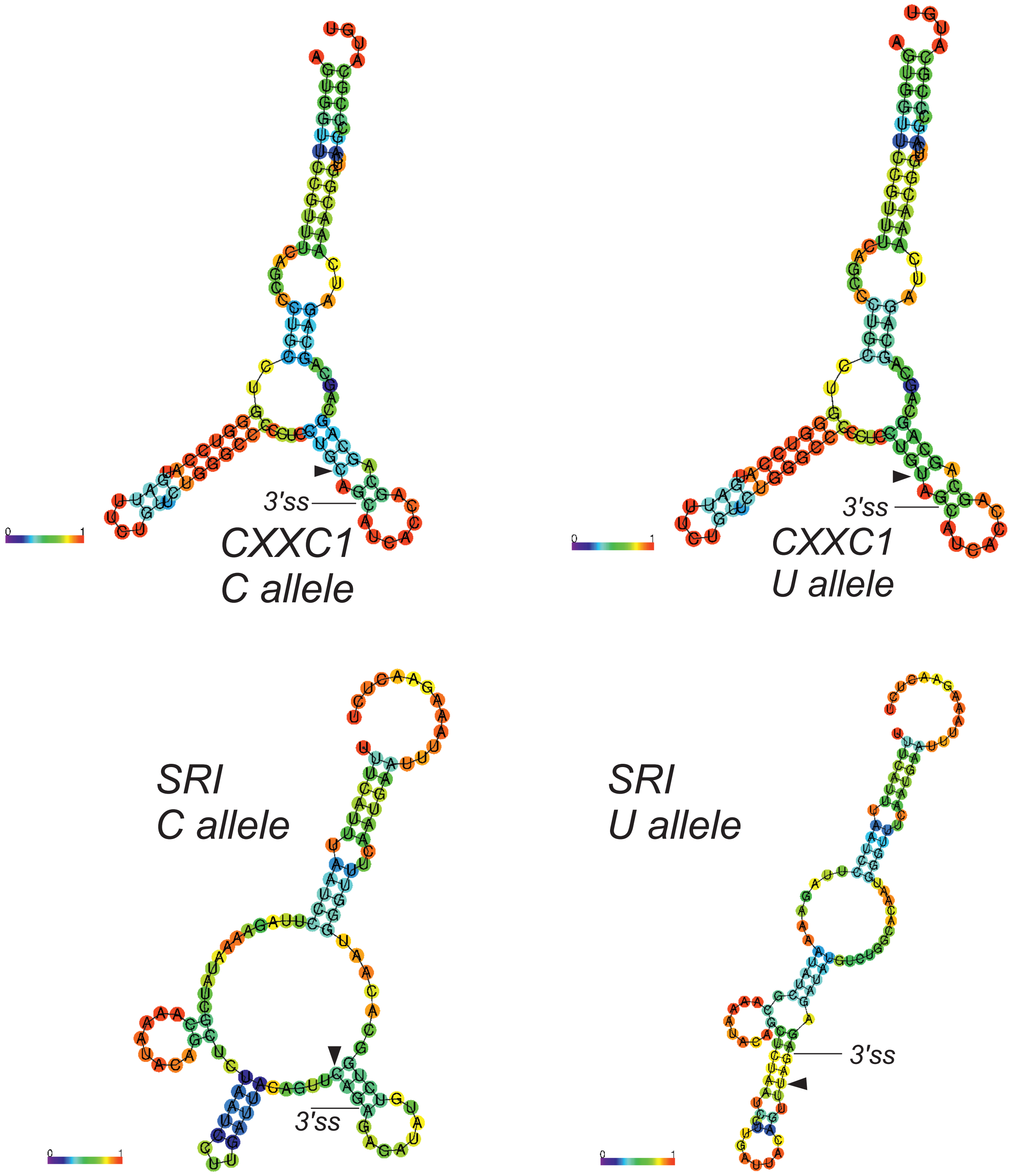
3'ss
CXXC1
C allele
0
1
3'ss
CXXC1
U allele
0
1
SRI
C allele
3'ss
0
1
SRI
U allele
3'ss
0
1

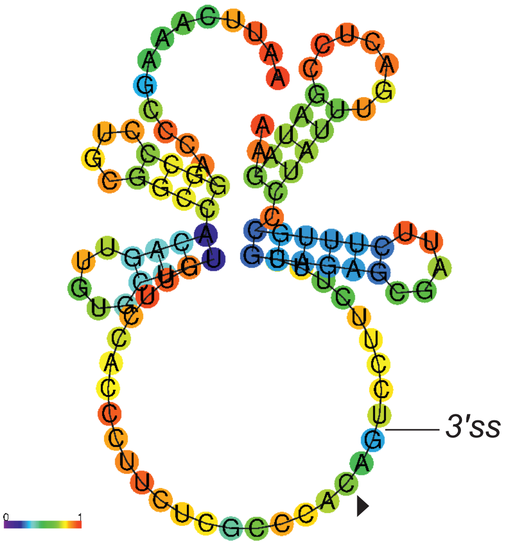

NOX5
C allele

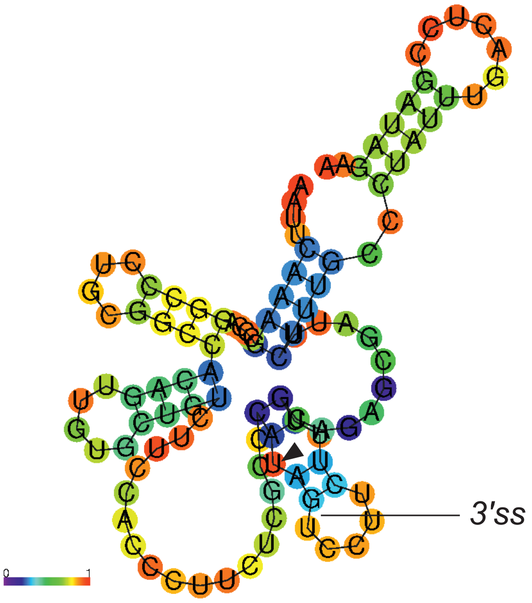

NOX5
U allele

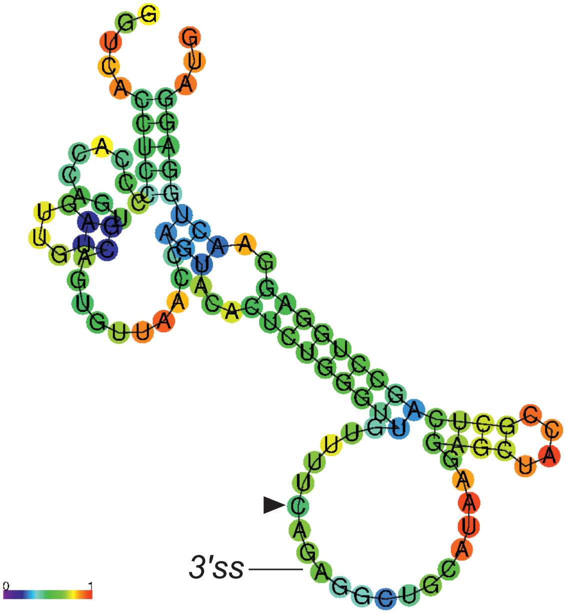

CRACR2A
C allele

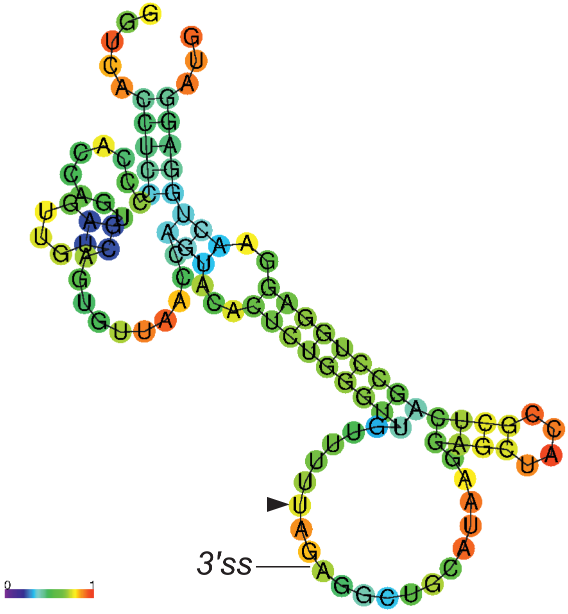

CRACR2A
U allele

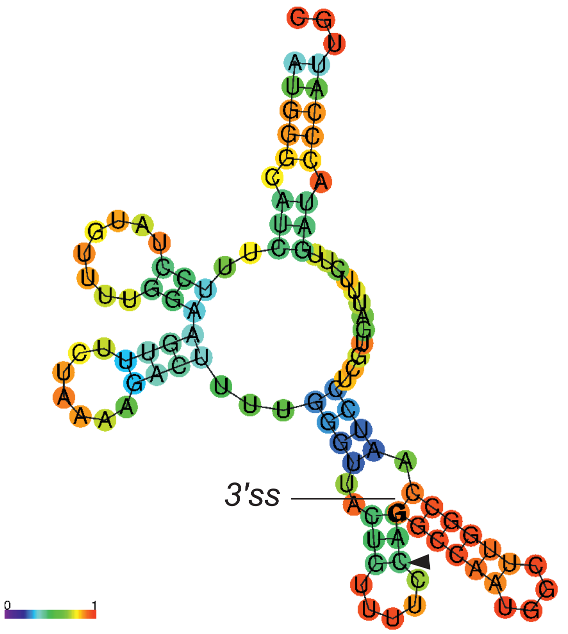

HGD
C allele

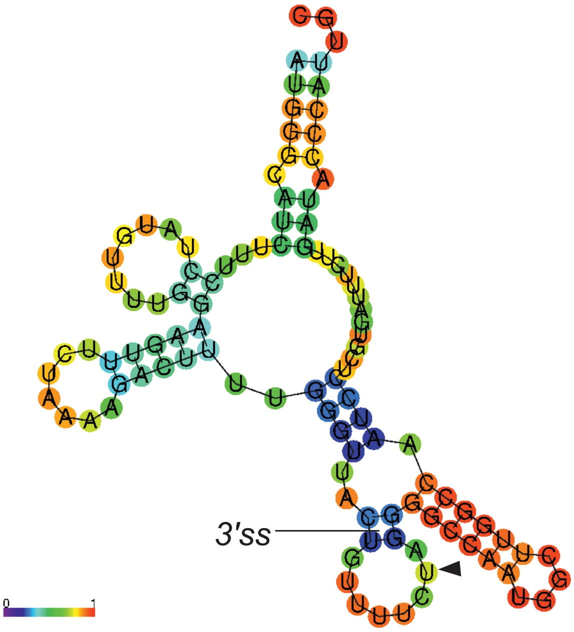

HGD
U allele

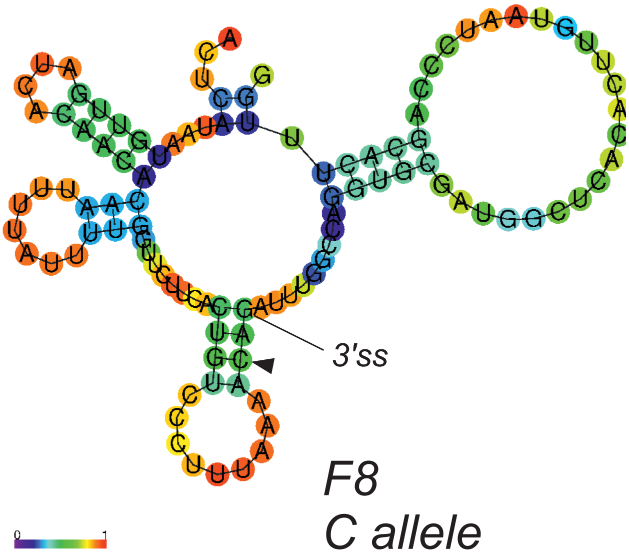

F8
C allele

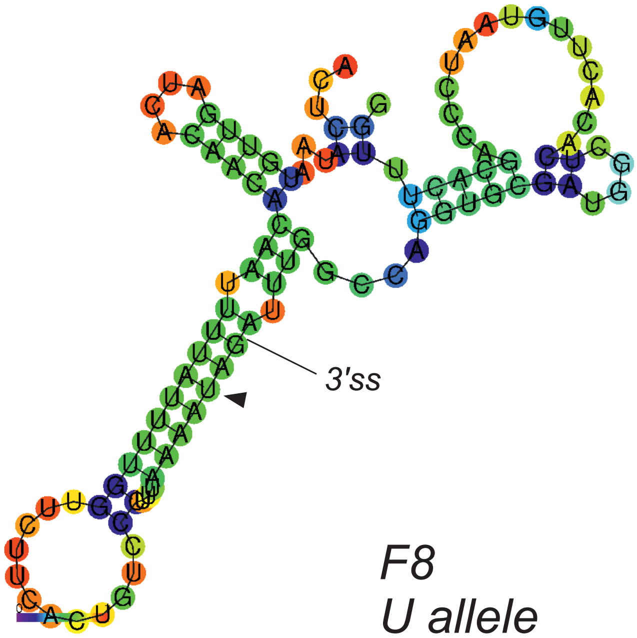

F8
U allele

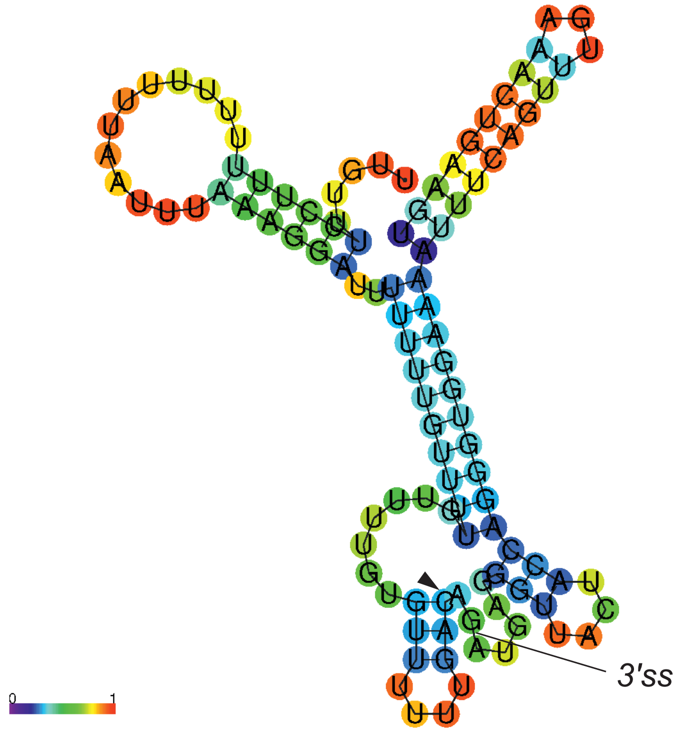

UBE2F
C allele

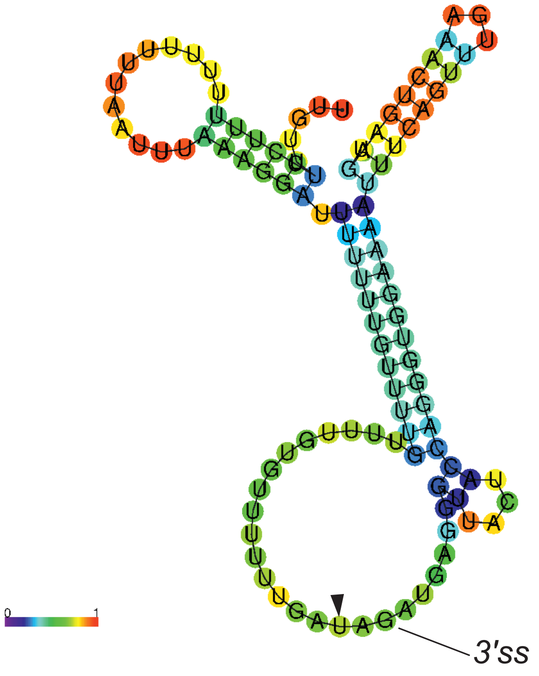

UBE2F
U allele

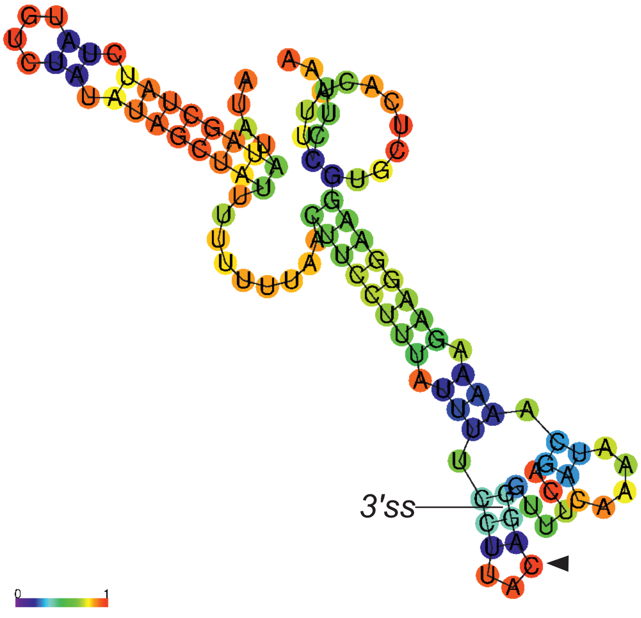

SMN1
C allele

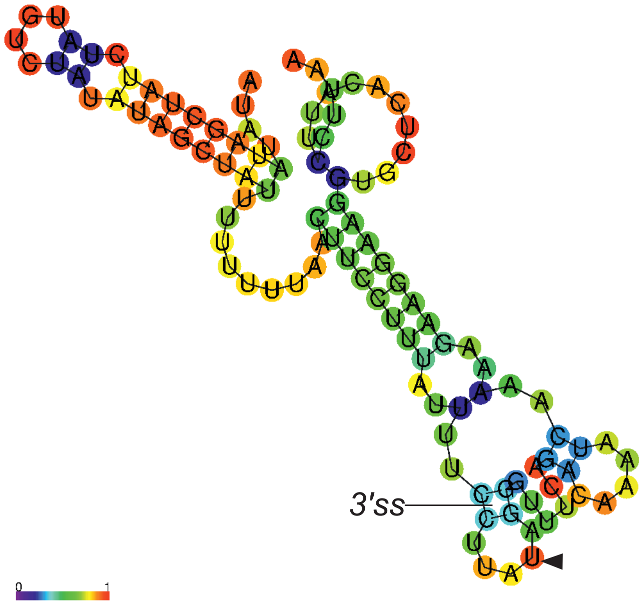

SMN1
U allele

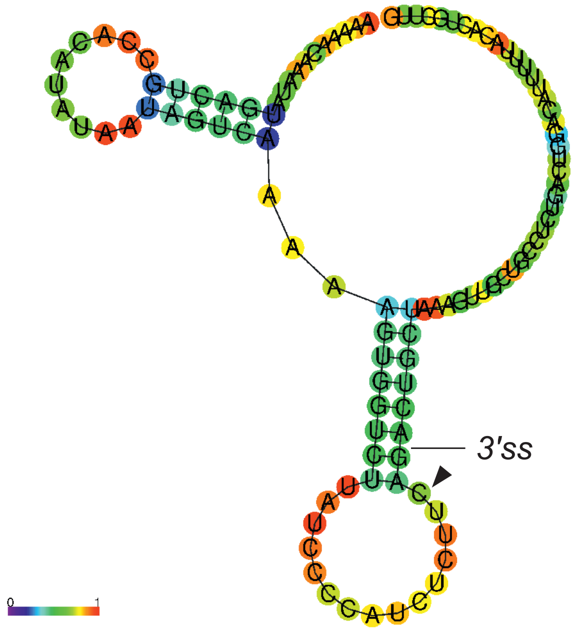

OTC
C allele

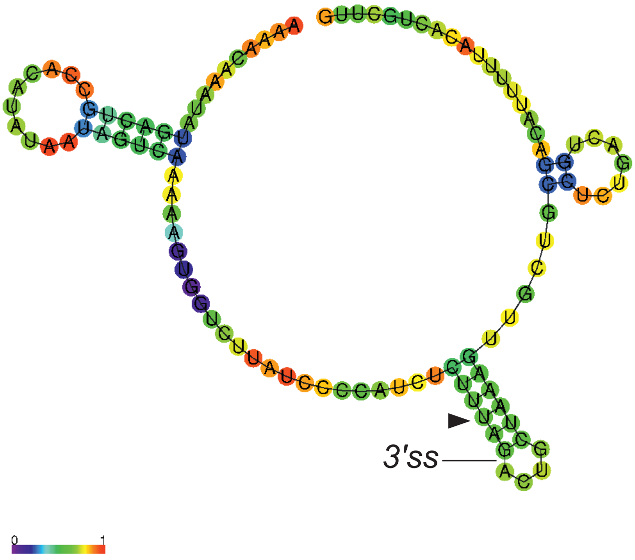

OTC
U allele

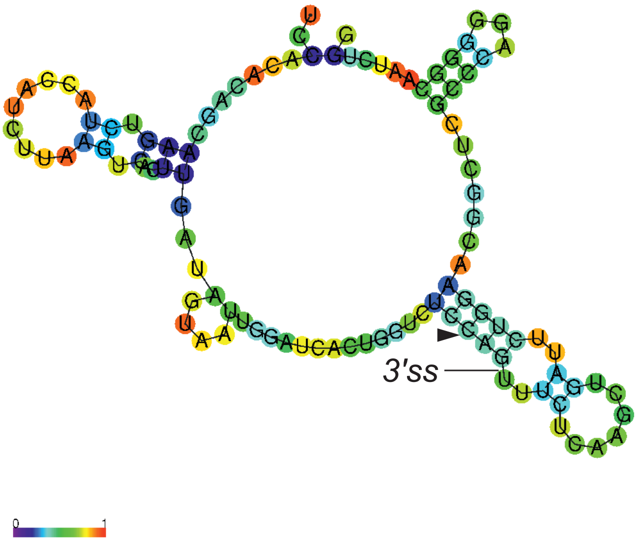

PKHD1
C allele

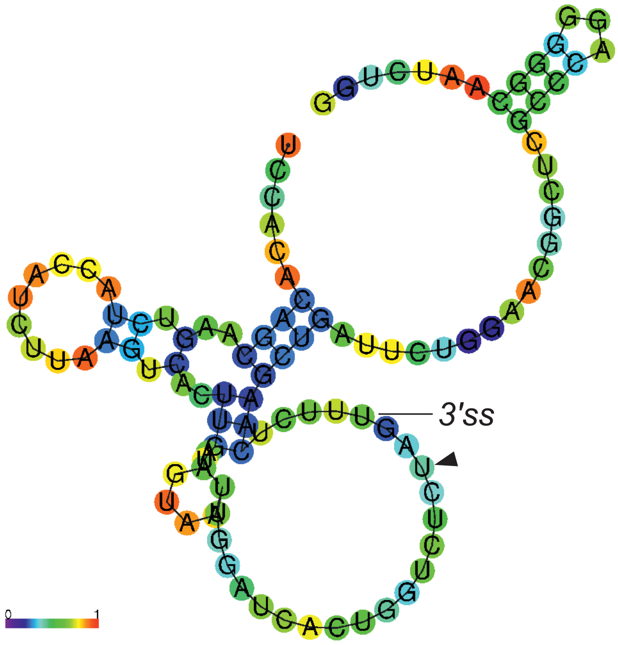

PKHD1
U allele

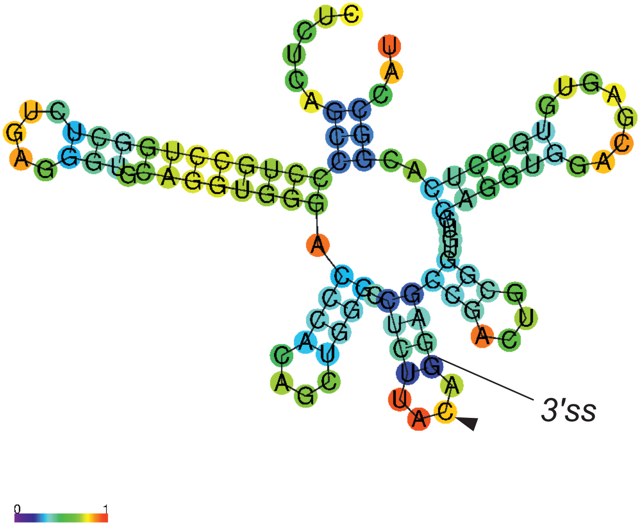

CRB2
C allele

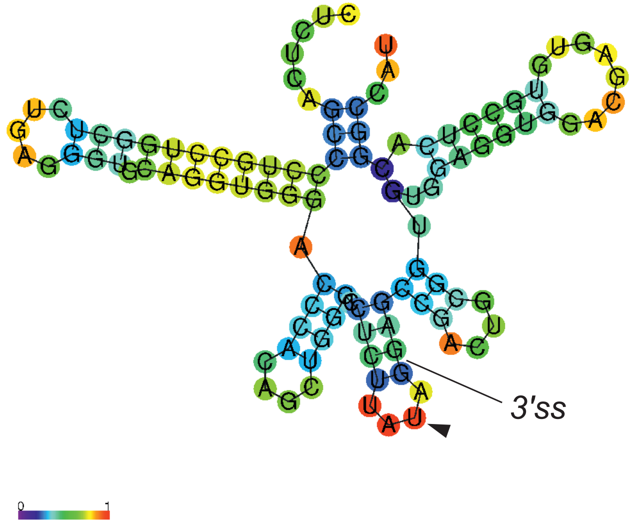

CRB2
U allele

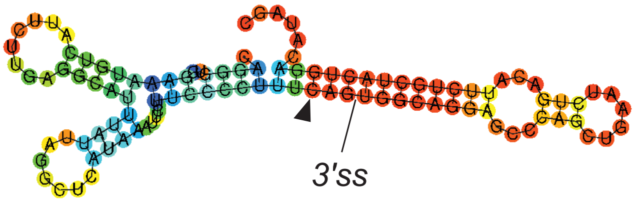

FRMD7
C allele

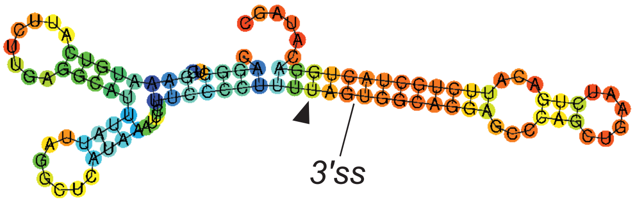

FRMD7
U allele

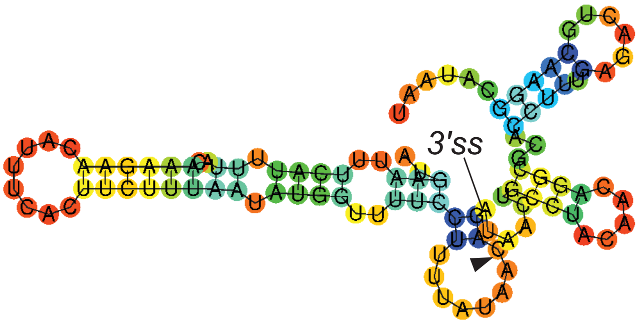

SGCE
C allele

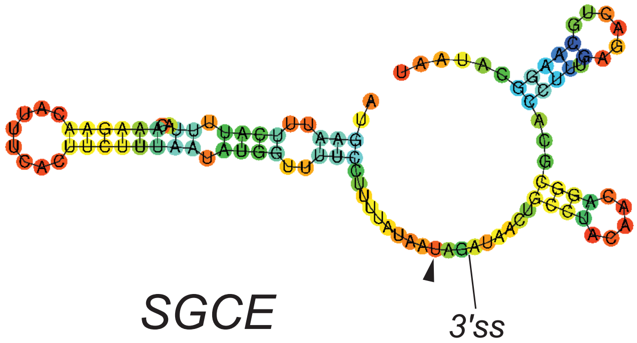

SGCE
U allele

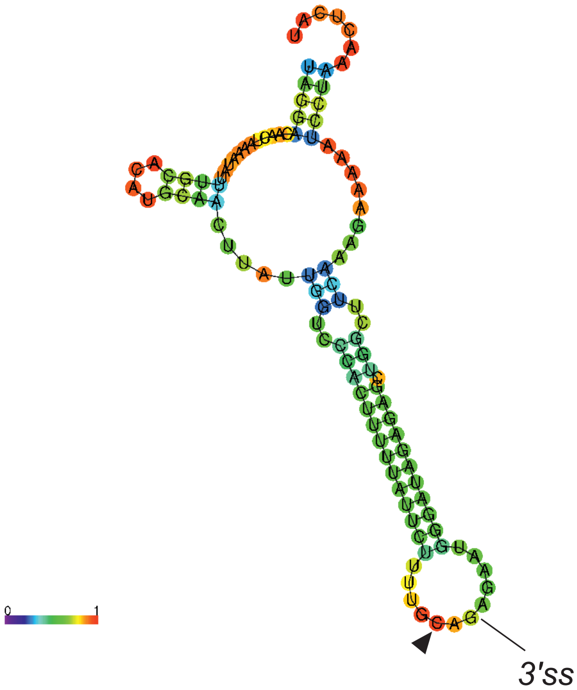

CFTR-E3
C allele

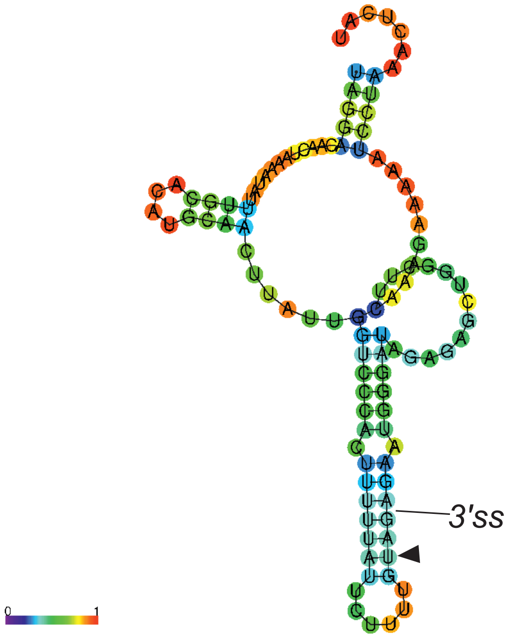

CFTR-E3
U allele

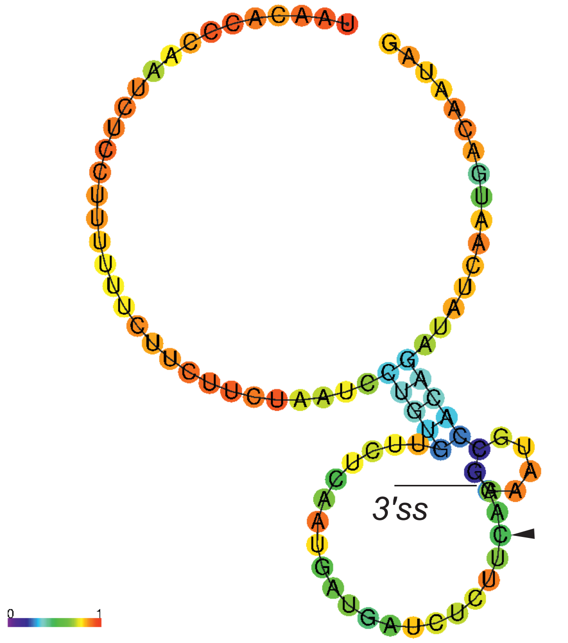

KIF5A
C allele

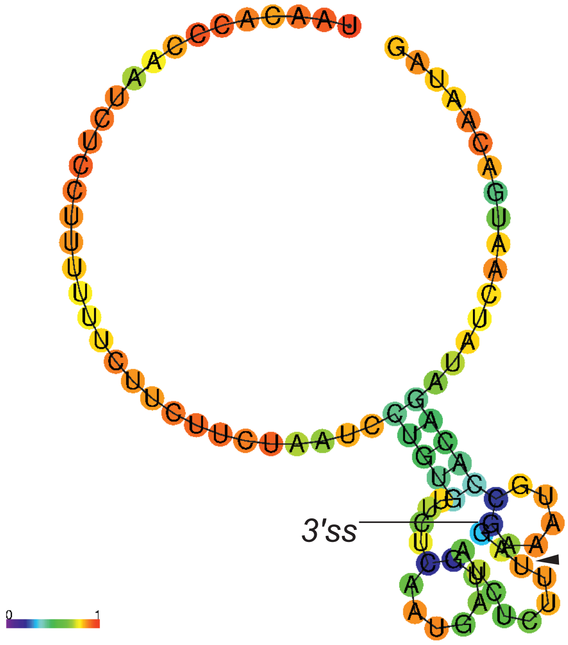

KIF5A
U allele

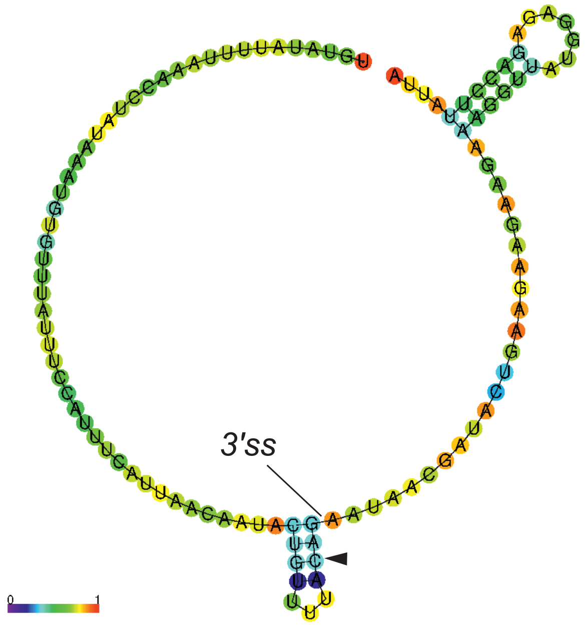

NIPBL
C allele

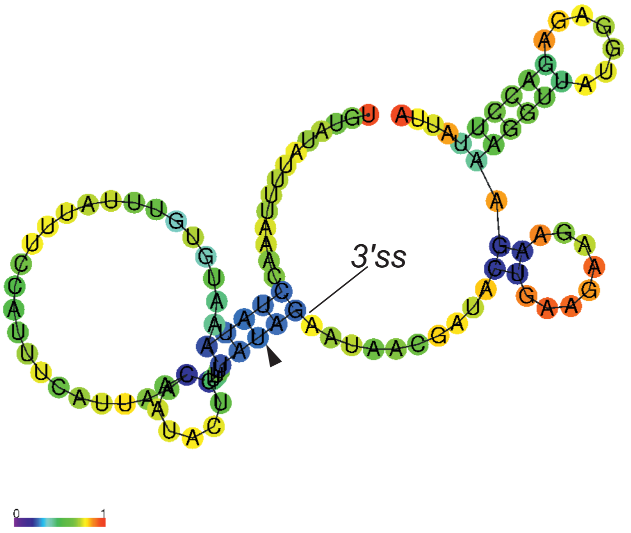

NIPBL
U allele

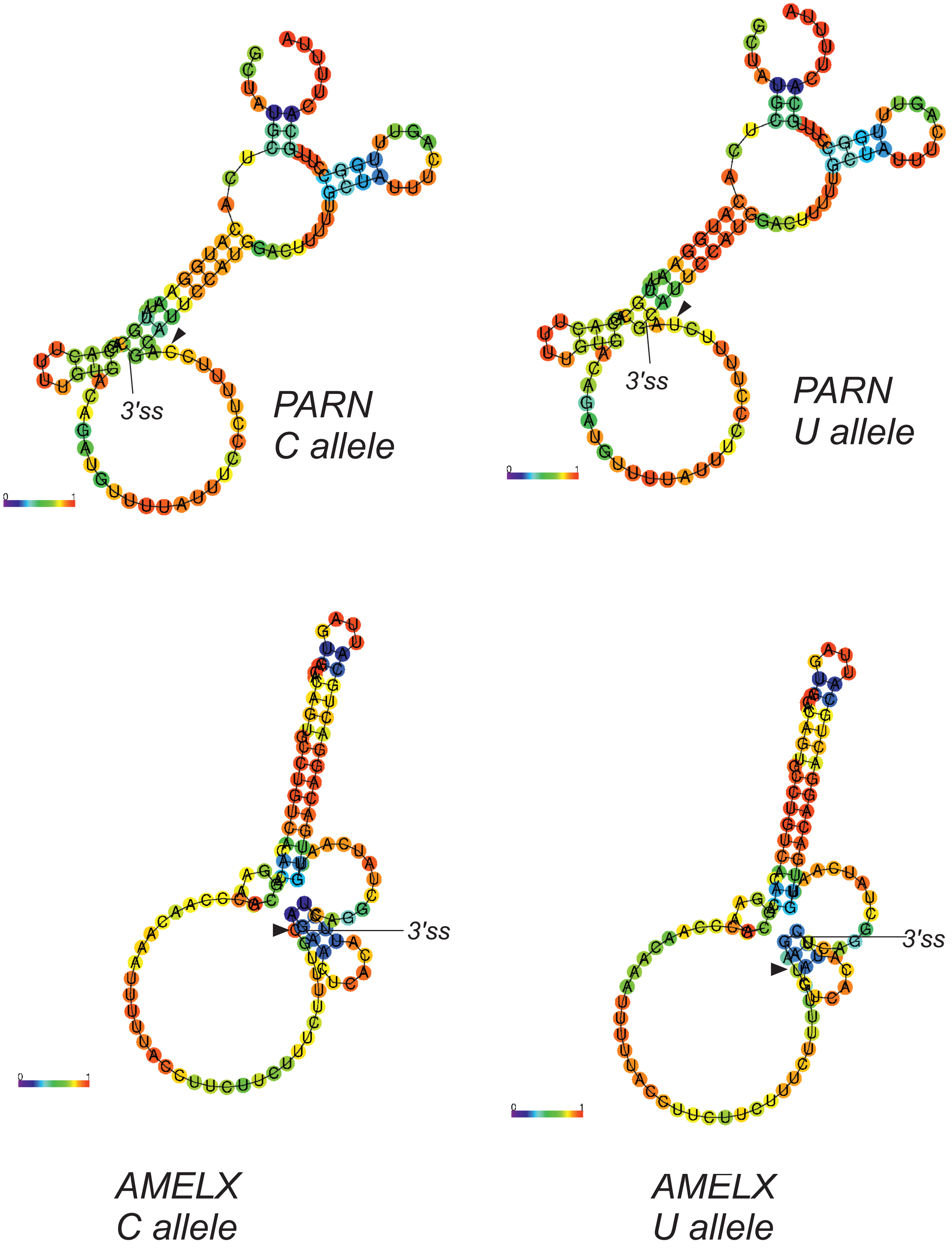
3'ss
PARN
C allele
3'ss
PARN
U allele
3'ss
3'ss
AMELX
C allele
AMELX
U allele
